# Ultimate Frisbee Players: Characteristics according to Their Competitive Level and Spirit of the Game

**DOI:** 10.3390/sports10120197

**Published:** 2022-12-02

**Authors:** José Pedro Amoroso, João Valente-dos-Santos, Guilherme Eustáquio Furtado, Ricardo Rebelo-Gonçalves, Raul Antunes, Luís Calmeiro

**Affiliations:** 1 Polytechnic Institute of Leiria, 2411-901 Leiria, Portugal; jose.amoroso@ipleiria.pt (J.P.A.); ricardo.r.goncalves@ipleiria.pt (R.R.-G.); 2 CIEQV-Life Quality Research Center, 2040-413 Leiria, Portugal; 3 CIDEFES—Centro de Investigação em Desporto, Educação Física e Exercício e Saúde, Lusófona University, 1749-024 Lisboa, Portugal; joao.valente.santos@ulusofona.pt; 4 Research Unit for Sport and Physical Activity (CIDAF–uid/dtp/04213/2020), University of Coimbra, 3040-156 Coimbra, Portugal; guilhermefurtado@ipc.pt or furts2001@yahoo.com.br; 5 Polytechnic Institute of Coimbra, Applied Research Institute, Rua da Misericórdia, Lagar dos Cortiços–S. Martinho do Bispo, 3045-093 Coimbra, Portugal; 6 Center for Innovative Care and Health Technology (ciTechCare), Polytechnic of Leiria, 2410-541 Leiria, Portugal; 7 School of Applied Sciences, Abertay University, Dundee DD1 1HG, UK; l.calmeiro@abertay.ac.uk; 8 Faculty of Medicine, Institute of Environmental Health, University of Lisbon, 1649-028 Lisboa, Portugal

**Keywords:** teamwork, sportsmanship, self-refereeing, competition, flying disc

## Abstract

In Ultimate Frisbee, players are responsible for administering and reinforcing adherence to the rules of the game. As a self-refereed sport, UF relies upon the Spirit of the Game (SOTG). This study aimed to profile the experience, to ascertain the training history of the sport, the participation and goal orientations of international Ultimate Frisbee players in the European Beach Ultimate Championship, and to evaluate the contribution of these variables in a discriminant function to classify players according to SOTG level and competitive level. The sample consisted of 160 players [females 33.8% (29.3 ± 7.2 years); males 66.2% (37.6 ± 9.7 years)] who competed in the European Beach Ultimate Championship, 2019. Factorial ANOVA was used to test the effect of sex, competitive level and SOTG level (measured by the sum of the scores obtained in five questions addressing the following domains: (1) Knowledge and use of the rules; (2) Fouls and body contact; (3) Fair-mindedness; (4) Positive attitude and self-control; (5) Communication. The results revealed that sex was not a consistent source of SOTG level variation among players. In each competitive level, those with high SOTG levels obtained lower European Beach Ultimate Championship classification (t = 5.73 to 6.55, *p* < 0.001, d = 1.28 to 2.06), higher SOTG classification (t = −13.21 to −7.04, *p* < 0.001, d = 1.28 to 2.85), and better evaluation for fouls and body contact (t = 2.76 to 9.23, *p* < 0.05, d = 0.86 to 1.99) and for positive attitude (t = 5.88 to 7.43, *p* < 0.001, d = 2.06 to 2.57), than regular SOTG level players. Players of different competitive levels demonstrated similar SOTG results. These findings provide important information to coaches, physical education teachers and sport consultants.

## 1. Introduction

Ultimate Frisbee (UF) is a fast paced, non-contact, mixed-gender team sport played with a flying disc or frisbee [1]. In its three versions, the game is played by two teams of seven players on grass, five players on the beach (Beach Ultimate) and four players indoors. Gathering attributes of several invasion games, such as American football and netball, into a simple yet demanding game [2], UF is a self-refereed game which requires players to give up possible illicit advantages. Hence, UF has distinguishing features when compared to other team sports, such as self-arbitration, and independent communication, even at a world championship level, where players are expected to abide by a code of fair play, known as the Spirit of the Game (SOTG) [3].

The SOTG can largely be built from the following five dimensions: rules knowledge, fouls and body contact, fair-mindedness, positive attitude, and communication. These dimensions are used in the process of self-evaluation and opponent evaluation during official WFDF tournaments. The purposeful practice of each of these elements drives the growth of spirited players and teams. The SOTG is touted as the feature that most sets UF apart from other competitive team sports, as it primarily upholds the doctrine of sportsmanship. UF players recognize that they did not invent sportsmanship, but they insist that UF is different in that it does more than “pay lip service” to the concept. The SOTG and the system of self-refereeing in UF seem to reflect the revival of a nineteenth-century ideal of sportsmanship and fair play [4]. The SOTG puts these ideals into practice, and to some extent, appears to modulate behaviors, actions, and some psychological aspects of the game [2].

Some aspects of SOTG, described since the beginning of UF [5], seem to encourage researchers to seek an understanding of the psychological dimension of UF [1,6,7]. For example, promoting highly competitive play but never at the expense of mutual respect among players, playing for pleasure and joy, and not endorsing actions such as provoking opponents, encouraging intentional aggressions or even “win at all costs” behaviors, are unique to UF; therefore, it is argued that UF is an ideal context to promote sportsmanship behavior.

The social context of sport provides interaction experiences, which are particularly relevant in team sports [8]. Coaches and players should pursue high collective efficacy standards within their team. These social interactions between coaches and players may also offer interesting insights into the UF atmosphere, within which a single person may perform multiple roles. These roles comprise a variety of dimensions, such as evaluative, training and competitive roles, and roles associated with the meeting of social and emotional needs [9]. Such a variety of roles creates a rich environment for the development of unique social dynamics.

In competitive sports, the importance placed on outcomes toward the end of the season increases. It is in the latter stages of the season that promotion, relegation, and championship places are decided [8]. Therefore, the cost of winning and losing is likely to result in stronger ego-involvement and weaker perceptions of a mastery motivational climate. It, thus, becomes important to measure goal orientation in the context of sport, in order to identify which players are more ego-oriented and which are more task-oriented [10]. To avoid this happening, findings suggest that coaches should stress the importance of enthusiasm and communication, and train their players to continue communicating, even when the team is losing [11].

SOTG encourages UF players’ positive interaction by emphasizing communication and team cooperation on the field [12]. The interpretation of SOTG varies with the level of competition, history of games between the same teams and experience of the players.

It is, therefore, essential to characterize the players of this sport, in order to better understand how some variables, behave and to identify differentiating aspects that may be important for coaches and teachers to consider [7]. It is also important to increase the number of studies in this sport and consider other practice contexts [13]. In addition, the literature has reinforced the important role of alternative sports as tools for promoting the global health of subjects at different ages [14,15].

Based on this theoretical framework, this study was intended to profile the experience, training history, European Beach Ultimate Championship (EBUC) participation, SOTG and goal orientations of international UF players. In addition, the central objective of this study was to evaluate the contribution of these variables to the discrimination of players by SOTG level (i.e., high and regular) in the total sample and by competitive level (i.e., highest, intermediate, and lowest).

## 2. Materials and Methods

### 2.1. Participants

**Figure 1:**
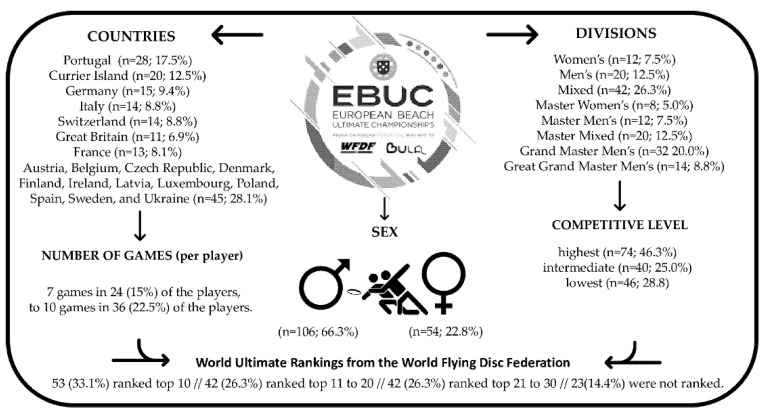
Summary of participants characteristics.

The present study comprised a total sample of 160 players: 33.8% were females (29.3 ± 7.2 years of age) and 66.2% were males (37.6 ± 9.7 years of age) participating in the EBUC, 2019, held in Portimão, Portugal, from 6th to 11th of May, 2019. Characteristics of the players participating in the present study are summarized in Figure 1. Nineteen European countries were represented in eight divisions (women’s, men’s, mixed, master women’s, master men’s, master mixed, grand master men’s and great grand master men’s) across three different competitive levels (highest vs. intermediate vs. lowest). Competitive levels were determined by the World UF rankings from the WFDF: 33.1% were ranked top 10; 26.3% were ranked top 11 to 20; 26.3% were ranked 21 to 30 and (14.4%) were not ranked. Figure 1 also presents the number of games played by each player during the Championship.

Cross-tabulations of UF players’ SOTG levels, by sex and competitive level, are summarized in Table 1. Data indicated a similar percentage of male and female players (χ^2^ = 3.20, *p* = 0.07) and a similar percentage of highest, intermediate, and lowest competitive level players (χ^2^ = 2.96, *p* = 0.23) per SOTG level (two clusters, i.e., high vs. regular, developed based on principal component analysis and hierarchical classification), according with World UF Ranking from the WFDF.

### 2.2. Instrumentation

#### 2.2.1. Spirit of the Game Scoring System

The system was designed in accordance with the expectation that teams generally display normal, good Spirit. Therefore, the baseline in each category is “Good”, which equals 2 points. The self-referenced system is evaluated quantitatively for the assignment of the SOTG award. During SOTG scoring the teams are advised to discuss each category separately. SOTG is measured, based on a marking system used immediately after each game. Players assess the SOTG of the opposing team and their own team according to the five principles of the game. SOTG is measured by the sum of the scores obtained in five questions addressing the following domains: (1) Knowledge and use of the rules; (2) Fouls and body contact; (3) Fair-mindedness; (4) Positive attitude and self-control; (5) Communication. Answers are given in a 5-point Likert scale (0 = Poor; 1 = Not Good; 2 = Good; 3 = Very Good; 4 = Excellent). After each game, players rate if the other team was better than, worse than, or the same as a regular game, using the anchor “Good” as a baseline for comparison. The final SOTG score is the sum of the answers to all questions and may vary between 0 and 25, where a score of 10 is considered normal, Good Spirit [1].

#### 2.2.2. Perception of Success Questionnaire

Participants were asked to respond to the Perception of Success Questionnaire (POSQ) [16], which included 12 items (e.g., “when playing ultimate frisbee, I feel most successful when…”) measured on 5-point Likert scale (ranging from A = strongly agree to E = strongly disagree). The confirmatory factor analyses performed by Roberts et al. [16] revealed that the POSQ is a reliable and valid instrument to measure achievement goal orientations, in the context of sport and physical activity, both in adults and in children. Data obtained through the sociodemographic questionnaire information and practice-related information were analyzed, including data on sex, age, UF experience, national team experience, weekly training sessions and volume of same, country world ranking, EBUC games played and classification and POSQ, in the total sample and by competitive level.

### 2.3. Procedures

A sociodemographic questionnaire was distributed by the “Spirit Director” to all “Spirit of the Game captains” in the protocol meeting, one day before the beginning of the tournament. Participants were asked to complete the questionnaires alone and in a quiet environment. Instructions on how to complete the questionnaire were provided, emphasizing that responses would be kept confidential, and that answers should be as honest and spontaneous as possible. It was stressed that there were no right or wrong answers. The questionnaires required approximately five minutes to be completed. Due to the specific rules of the UF, we chose to use English as a standardized language for all participants in the questionnaires. To standardize the procedures, we also used English qualifications, and the Common European Framework of reference for languages. The study was conducted in accordance with recognized ethical standards for research in sports sciences [17] and approved by the Ethics Committee of the Faculty of Physical Education and Sport, Lusófona University, on 6 February, 2019, with report number F0619, and followed the Declaration of Helsinki produced by the World Medical Association for research with humans. Participants were fully informed about the nature of the study. Participation was voluntary and the right to withdraw from the study was explained. Written informed consent was obtained individually from each participant who agreed to participate in this study. In addition, permission was requested for the organization of the competition, so that data could be collected from the participating teams. Spirit Scoring is especially recommended for leagues and larger tournaments. In these events, a team’s Spirit Captain was responsible for collecting Spirit Scores and giving them to the Spirit Director. Subsequently, this information was received by, and sent on by, the volunteers to the tournament’s spirit director, who collected all the game data and saved it in an excel sheet of the EBUC.

### 2.4. Study Design

The present work was a cross-sectional study [18], and, specifically, a comparative study, as these are studies that analyze the relationship between variables by examining the differences that exist between two or more groups of individuals.

### 2.5. Principal Component Analysis and Hierarchical Classification

Principal component analysis was used to create a SOTG level profile, where rules knowledge, fouls and body contact, fair-mindedness, positive attitude, and communication were reduced into a single factor, using Varimax rotation with Kaiser Normalization. Adequacy was checked using the Kaiser–Mayer–Olkin index and Bartlett’s sphericity test. Based on a screen plot, an Eigenvalue > 1 and interpretability, factors from the first principal component were designated as the SOTG level profile. Higher scores corresponded to higher levels of SOTG. Based on the SOTG level profile, hierarchical classification techniques were employed for the classification and discrimination of the UF players into two clusters, i.e., high and regular SOTG levels, using Euclidean distance with Ward’s algorithm.

### 2.6. Statistical Analysis

Descriptive statistics were calculated. Factorial ANOVA was used to test the differences in the total sample between the following: (a) female and male players; (b) highest vs. intermediate vs. lowest competitive levels, and (c) high vs. regular SOTG level in sport experience, EBUC participation and goal orientations. The effect size correlations (ES-r) were estimated using the square root of the ratio of the t-value squared and the difference between the t-value squared and degree of freedom [19]. Coefficients were interpreted as follows: trivial (r < 0.1), small (0.1 < r < 0.3) moderate (0.3 < r < 0.5), large (0.5 < r < 0.7), very large (0.7 < r < 0.9), nearly perfect (r > 0.9) and perfect (r = 1).

Comparisons between high and regular SOTG level players by competitive level were performed using Student *t*-tests and standardized differences between means were reported using Cohen’s d values, interpreted as follows: <0.20 (trivial), 0.20 to 0.59 (small), 0.60 to 1.19 (moderate), 1.20 to 1.99 (large), 2.0 to 3.9 (very large), and >4.0 (extremely large) [20]. Pearson’s product moment correlation coefficients were calculated to examine the magnitude and direction of relationships, for the total sample, between high SOTG level and age, sport and training experience, national team world ranking, EBUC games played and classification and motivational climate, and by competitive level. The magnitude of correlations was interpreted as follows [20]: trivial (r < 0.1), small (0.1 < r < 0.3) moderate (0.3 < r < 0.5), large (0.5 < r < 0.7), very large (0.7 < r < 0.9), and nearly perfect (r > 0.9). Using variables that were significantly influenced by SOTG level, discriminant function analysis was utilized to obtain a predictive model that permitted classification of UF players as high and regular, i.e., the original groupings, for the total sample, and by competitive level. It was possible to order the predictors by the magnitude of correlations with the linear function. Subsequently, a stepwise model was used to test the hypothesis of extracting an alternative predictive model based on a smaller set of variables without losing explained variance. Significance was set at 5%. Statistical analyses were done with SPSS version 27.0 (SPSS Inc., IBM Company, Armonk, NY, USA).

## 3. Results

Descriptive statistics by sex, competitive level and SOTG level are summarized in Table 2. Sex was not a consistent source of variation among players (Table 3). Players differed significantly in competitive level in chronological age (CA), sport experience, training history, EBUC resulting variables and task orientation. The effect of the SOTG level was noted for CA, national team world ranking, EBUC games played and classification. The interaction of the SOTG level and the competitive level was a consistent source of variation among UF players, for all variables. Players of the highest competitive level divisions were, on average, younger and less experienced than intermediate and lowest level players, but reported a higher number of weekly training sessions and volume. The highest competitive level players also played more games during the EBUC and obtained a lower SOTG classification and SOTG score than the lowest competitive level players. Finally, the highest competitive players had a higher task orientation than the lowest competitive level players. High SOTG level players were significantly older, belonged to a national team with moderately lower world ranking, played fewer games during de EBUC and obtained a lower EBUC classification than regular SOTG level players.

In each competitive level, those with high a SOTG level obtained a lower EBUC classification, higher SOTG, better evaluations for fouls and body contact and positive attitude, than regular SOTG level players (Table 4). High SOTG level players also had less UF and national team experience, higher weekly training volume, belonged to teams with lower world ranking, played less games in the EBUC and obtained higher scores in fair-mindedness and communication. Players of contrasting SOTG level at each competitive level did not differ in rules knowledge and task orientation. Ego orientation was significantly lower in high SOTG level players of the lowest competitive level.

Table 5 summarizes the interrelationships between high SOTG level with age, UF experience, national team experience, weekly training sessions and volume, country world ranking, EBUC games played and classification, in the total sample, and by competitive level.

The stepwise protocol used in the discriminant analysis indicated a linear combination of three variables that differed between high and regular SOTG level players (Table 6). The final model included EBUC classification, EBUC games played and weekly training sessions. The analysis by competitive level also successfully distinguished high and regular SOTG level players. The EBUC classification was identified as a common predictor for each competitive level.

## 4. Discussion

The purpose of this study was to examine the experience, training history, European Beach Ultimate Championship (EBUC) participation and goal orientations of international UF players, and to evaluate the contribution of these variables in a discriminant function to classify players according to SOTG level and competitive level. The effect of SOTG level was particularly observed for CA, national team world ranking, EBUC games played and classification.

The classification was identified as a common predictor for each competitive level. Those with high SOTG level obtained a lower EBUC classification. In addition, the highest competitive players had a higher task orientation than the lowest competitive level players. Moreover, ego orientation was significantly lower in high SOTG level players of the lowest competitive level.

The interpretation of the SOTG varied with the level of competition, history of games between the same teams and the experience of the players. In accordance with Amoroso et al. [7], players of the highest competitive level divisions were, on average, younger and less experienced than intermediate and lowest level players but reported a higher number of weekly training sessions and volume.

The highest competitive level players played more games during the EBUC and obtained a lower SOTG classification and SOTG score than the lowest competitive level players. Players with the highest SOTG scores were also the ones who reported fewer fouls and body contact, were fairer, had more positive attitudes, and better communication. UF and SOTG promotes those behaviors and guides the development of an approach to participation that enables youngsters in daily training to define sports competitions with focus and good direction [7].

In each competitive level, those with high SOTG levels obtained a lower EBUC classification, higher SOTG classification, and better scores for fouls and body contact, and positive attitude, than regular SOTG level players. Ego-orientation was significantly lower in high SOTG players in the lowest competitive level than in regular SOTG players. Considering that the pursuit of ego goals may promote cheating in sport, these results provided strong empirical support for ego-orientation being inversely associated with the spirit of the game, in line with the literature of moral reasoning and moral behavior in sport [21].

The stepwise protocol used in the discriminant analysis indicated a linear combination of three variables that differed between high and regular SOTG level players. The final model included EBUC classification, EBUC games played and weekly training sessions, suggesting that those who had more contact with the sport had a “better spirit”. High SOTG level players obtained a significantly higher SOTG classification derived from a higher score on four of the five dimensions (i.e., high SOTG players committed fewer fouls, had less body contact, were fairer, and demonstrated greater positive attitude and better communication).

In this study, players of different competitive levels demonstrated similar SOTG results. Intimidation, intentional fouling, or other ‘‘win-at-all-costs’’ behavior are contrary to the spirit of the game and must be avoided by all players [22]. The analysis by competitive level also successfully distinguished high and regular SOTG level players. The EBUC classification was identified as a common predictor for each competitive level.

Our findings indicated that sex was not a consistent source of variation among players, which could suggest gender equity among UF players regarding their sport experience, training history, EBUC resulting variables, and goal orientation. Policies seeking to promote gender equity in sport need to enforce changes in club environments in addition to focusing on increasing women’s participation [23]. All over Europe, integrating a gender perspective to enhance sport participation is one of the most prominent targets of sport policy today, since research shows that men participate more often in sports than women [24].

Players differed significantly by competitive level in CA, sport experience, training history, EBUC resulting variables and task orientation, training, and competition contexts. These could be considered when researchers investigate achievement motivation in sport, and application of the rules of safe play, fair-mindedness, calm communication, and positive and respectful attitude to protect the basic joy of play. This transformation is delicate, and not automatic, and is certainly not guaranteed for all instances of gameplay. We should seek a changeable attitude towards ends, and we should seek well-designed games that enable the transformation of competition into cooperation [25].

**Figure 2:**
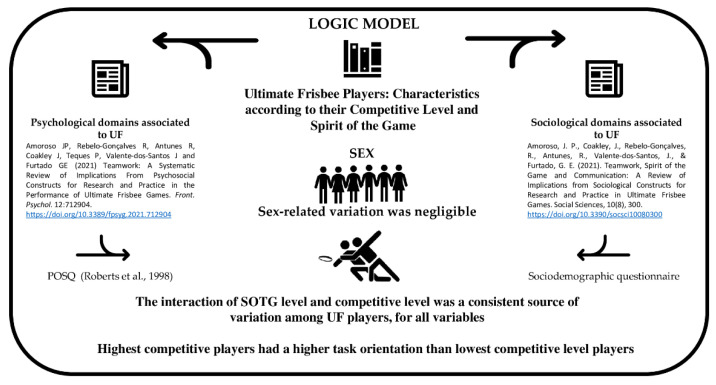
Summary of logic model [7,12].

The interaction of SOTG level and competitive level was a consistent source of variation among UF players, with improvements being obtained in all of them [26]. Siedentop [27] proposed the development of a competent, literate, and enthusiastic sportsperson as the main goals of sport education. Literacy encompasses the ethical dimension of the model. Therefore, it is the goal most closely related to the research variables of the present study: SOTG and competition levels (Figure 2).

Although high SOTG level players obtained a significantly higher SOTG classification, notwithstanding controversial elements, it was suggested that being male or female did not interfere with ego or task orientation in SOTG variables [28]. The observance of fair play seemed to be most often equated with how the rules of the game were observed, with its emphasis on individual rights and responsibilities. It seemed to serve as a guide to the underlying assumptions philosophers make about the possibility of teaching appropriate moral behavior [29]. High and regular SOTG level players did not differ in rules knowledge. Individuals with high learning orientation tend to engage in more effortful cognitive processes when learning a new task or knowledge domain. In UF, self-refereeing creates a form of ideological social control, whereby rule violations and disputes are dealt with through a well-established ‘ritual’ of resolution between any two given players in a way that maximizes game flow [1].

## 5. Conclusions

In summary, to examine the experience, training history, and goal orientations of international UF players participating in the European Beach Ultimate Championship (EBUC), our results noted that sex was not a consistent source of SOTG score variation among players. On the other hand, the interaction of SOTG level and competitive level was a consistent source of variation among UF players, for all variables. Players differed significantly by competitive level in CA, sport experience, training history, EBUC resulting variables and task orientation. Ludic and competitive contexts should be considered when researchers investigate achievement motivation in sport. An effect of SOTG level was noted for CA, national team world ranking, EBUC games played and classification. The interaction of SOTG level and competitive level was a consistent source of variation among UF players, for all variables.

On average, players of the highest competitive level were younger and less experienced than intermediate and lowest competitive levels. However, they reported a higher number of weekly training sessions and volume of the same. Moreover, the highest competitive level players played more games during the EBUC and obtained a lower SOTG classification and SOTG score than the lowest competitive level players. Therefore, players who had more contact with UF had better SOTG values.

Despite the importance of the present study, some limitations must be acknowledged and should be addressed in future studies. First, the nature of the study did not allow us to draw great conclusions regarding the relationships established between the variables or the effect of the practice of the modality on the variables analyzed. Therefore, future studies should try to make an effort and analyze them longitudinally, so that causal relations can be tested. Secondly, the data collection was made at the same event, and the players’ perceptions may have been influenced by contextual variables related to the event. It would be important in future studies, in addition to collecting larger samples, to be able to access different events of this alternative sport.

The findings provide important information to coaches, PE teachers and sport consultants, and may be of use in formulating SOTG preparation programs that could foster the experience of sportsmanship and facilitate the ethical conduct of players, in either ludic or competitive contexts. Using SOTG requires that every player knows the rules. Players are responsible for their behavior and for self-refereeing, which is arguably a useful tool for children and adolescents to develop a sense of community through sport experience. This perspective may be useful to develop interventions that prepare players to be more conscientious of their actions and help teams to improve the spirit of the game.

## Figures and Tables

**Table 1:** Cross-tabulations (absolute and relative frequencies) of UF players’ SOTG level by sex (upper) and competitive level (lower).

	SOTG Level		χ^2^	df	*p*
	High	Regular			
Sex:					
Female	30 (18.75%)	24 (15.0%)	3.196	1	0.074
Male	74 (46.25%)	32 (20.0%)			
Competitive Level:					
Highest	43 (26.88%)	31 (19.38%)	2.955	2	0.228
Intermediate	29 (18.13%)	11 (6.88%)			
Lowest	32 (20.0%)	14 (8.75%)			
Total	104 (65.0%)	56 (35.0%)			

UF (Ultimate Frisbee); SOTG (Spirit of the Game).

**Table 2:** Means and standard deviations for all variables as a function of sex, competitive level and SOTG level.

	Sex		Competitive Level			SOTG Level	
	Female (n = 54)	Male (n = 106)	Highest (n = 74)	Intermediate (n = 40)	Lowest (n = 46)	High (n = 104)	Regular (n = 56)
Chronological age, years	29.3 ± 7.2	37.6 ± 9.7	26.2 ± 3.8	37.5 ± 4.4	45.9 ± 5.3	36.5 ± 9.7	32.7 ± 9.4
UF experience, years	7.3 ± 3.0	8.5 ± 2.9	6.6 ± 2.8	9.8 ± 2.0	9.2 ± 2.8	8.0 ± 3.1	8.3 ± 2.9
National team experience, years	3.3 ± 3.1	4.0 ± 3.6	2.6 ± 2.2	4.5 ± 3.7	4.8 ± 4.2	3.3 ± 3.1	4.5 ± 3.8
Weekly training sessions, ^#^	2.1 ± 0.8	2.0 ± 0.8	2.4 ± 0.8	1.7 ± 0.6	1.6 ± 0.6	1.9 ± 0.7	2.1 ± 0.8
Weekly training volume, min	204 ± 87	187 ± 85	237 ± 82	160 ± 70	152 ± 70	186 ± 80	202 ± 94
Country world ranking, ^#^	14.1 ± 9.0	15.2 ± 9.2	14.9 ± 9.2	14.2 ± 8.8	15.2 ± 9.5	17.3 ± 9.3	11.1 ± 7.5
EBUC games played, ^#^	8.7 ± 1.0	8.5 ± 1.0	9.1 ± 0.9	8.6 ± 0.8	7.8 ± 0.7	8.4 ± 0.9	8.9 ± 1.0
EBUC classification, ^#^	6.5 ± 4.2	6.8 ± 3.4	7.5 ± 3.8	6.6 ± 3.8	5.6 ± 3.2	8.2 ± 3.3	4.6 ± 3.1
SOTG classification, ^#^	6.7 ± 5.2	5.8 ± 4.2	7.4 ± 5.5	5.7 ± 4.0	4.4 ± 2.4	3.5 ± 1.8	9.8 ± 4.7
Rules knowledge, ^†^	2.02 ± 0.09	1.99 ± 0.12	2.02 ± 0.09	2.04 ± 0.14	1.94 ± 0.11	2.01 ± 0.12	1.99 ± 0.11
Fouls and body contact, ^†^	1.95 ± 0.16	2.03 ± 0.21	1.97 ± 0.16	2.04 ± 0.31	2.04 ± 0.13	2.11 ± 0.16	1.87 ± 0.17
Fair-mindedness, ^†^	2.25 ± 0.19	2.33 ± 0.22	2.24 ± 0.22	2.33 ± 0.22	2.37 ± 0.19	2.41 ± 0.16	2.15 ± 0.20
Positive attitude, ^†^	2.58 ± 0.22	2.54 ± 0.20	2.48 ± 0.22	2.65 ± 0.18	2.57 ± 0.16	2.66 ± 0.14	2.39 ± 0.17
Communication, ^†^	2.19 ± 0.14	2.23 ± 0.19	2.27 ± 0.20	2.23 ± 0.19	2.13 ± 0.18	2.27 ± 0.18	2.13 ± 0.14
Total SOTG Score, ^‡^	10.98 ± 0.53	11.11 ± 0.57	10.97 ± 0.60	11.28 ± 0.63	11.06 ± 0.40	11.45 ± 0.32	10.53 ± 0.35
Task orientation, ^§^	4.46 ± 0.47	4.28 ± 0.67	4.52 ± 0.38	4.30 ± 0.64	4.08 ± 0.79	4.31 ± 0.64	4.36 ± 0.60
Ego orientation, ^§^	3.05 ± 0.74	3.09 ± 0.88	3.17 ± 0.85	3.22 ± 0.67	2.83 ± 0.87	3.01 ± 0.79	3.16 ± 0.88

SOTG (Spirit of the Game); UF (Ultimate Frisbee); EBUC (European Beach Ultimate Championship); ^#^ number, ranking or classification; ^†^ scale 0–4; ^‡^ sum of the results of the five categories; ^§^ scale 1–5;

**Table 3:** Results of ANOVA to test the main effects of sex, competitive level, and SOTG level, and the interaction effects of SOTG Level x Sex and SOTG Level x Competitive level.

	Effect of Sex			Effect of Competitive Level			Effect of SOTG Level			Interaction SOTG Level x Sex			Interaction SOTG Level x Competitive Level		
	F	*p*	ES-r	F	*p*	ES-r	F	*p*	ES-r	F	*p*	ES-r	F	*p*	ES-r
Chronological age	25.42	<0.001	0.40	256.86	<0.001 ^a^	0.89	5.27	0.02	0.19	9.34	<0.001	0.42	102.07	<0.001	0.14
UF experience	5.63	0.02	0.20	20.02	<0.001 ^b^	0.48	0.35	0.56	0.05	2.44	0.07	0.23	9.75	<0.001	0.05
National team experience	1.08	0.30	0.09	6.92	0.001 ^b^	0.31	3.99	0.05	0.17	2.68	0.05	0.24	5.39	<0.001	0.14
Weekly training sessions	1.03	0.31	0.09	20.10	<0.001 ^c^	0.48	2.05	0.16	0.12	2.11	0.10	0.21	9.05	<0.001	0.19
Weekly training volume	1.25	0.27	0.10	19.49	<0.001 ^c^	0.47	1.11	0.29	0.09	2.05	0.11	0.21	9.03	<0.001	0.17
Country world ranking	0.44	0.51	0.06	0.11	0.89	0.04	17.37	<0.001	0.34	7.13	<0.001	0.37	4.27	0.001	0.21
EBUC games played	1.02	0.31	0.09	28.21	<0.001 ^d^	0.54	10.48	0.002	0.27	3.80	0.01	0.28	13.53	<0.001	0.23
EBUC classification	0.18	0.67	0.04	3.66	0.03 ^e^	0.23	41.76	<0.001	0.49	14.04	<0.001	0.49	13.28	<0.001	0.48
SOTG classification	1.11	0.29	0.09	5.97	0.003 ^e^	0.29	118.63	<0.001	0.68	39.45	<0.001	0.69	28.53	<0.001	0.66
Rules knowledge	1.63	0.20	0.11	8.51	<0.001 ^f^	0.34	1.29	0.26	0.10	1.40	0.25	0.17	6.02	<0.001	0.16
Fouls and body contact	4.96	0.03	0.19	2.15	0.12	0.18	74.91	<0.001	0.60	25.98	<0.001	0.61	23.15	<0.001	0.69
Fair-mindedness	3.62	0.06	0.16	5.45	0.005 ^e^	0.27	67.02	<0.001	0.58	22.88	<0.001	0.58	22.30	<0.001	0.80
Positive attitude	1.06	0.31	0.09	9.01	<0.001 ^b^	0.34	103.36	<0.001	0.66	41.90	<0.001	0.70	26.21	<0.001	0.57
Communication	1.25	0.27	0.10	9.35	<0.001 ^f^	0.35	21.48	<0.001	0.37	7.15	<0.001	0.37	17.80	<0.001	0.68
Total SOTG Score	1.53	0.22	0.11	3.45	0.04 ^e^	0.22	258.02	<0.001	0.81	86.19	<0.001	0.81	71.46	<0.001	0.88
Task orientation	2.54	0.11	0.14	6.78	0.002 ^f^	0.30	0.22	0.64	0.04	0.90	0.44	0.14	2.95	0.02	0.16
Ego orientation	0.06	0.81	0.02	2.87	0.06	0.20	1.07	0.30	0.09	0.95	0.42	0.14	3.16	0.01	0.19

SOTG (Spirit of the Game); UF (Ultimate Frisbee); EBUC (European Beach Ultimate Championship). ^a^ Highest < Intermediate < Lowest. ^b^ Highest < Intermediate and Lowest. ^c^ Highest > Intermediate and Lowest. ^d^ Highest > Intermediate > Lowest. ^e^ Highest < Lowest. ^f^ Highest > Lowest.

**Table 4:** Means and standard deviations for all variables by SOTG level within competitive level.

Competitive Level	Highest (n = 74)		t	*p*	d	Intermediate (n = 40)		t	*p*	d	Lowest (n = 46)		t	*p*	d
SOTG Level	High (n = 43)	Regular (n = 31)				High (n = 29)	Regular (n = 11)				High (n = 32)	Regular (n = 14)			
Chronological age, years	26.3 ± 3.5	25.6 ± 3.7	0.75	0.45	0.18	37.7 ± 4.5	37.1 ± 3.5	0.40	0.69	0.14	46.8 ± 5.3	45.1 ± 5.4	1.04	0.31	0.32
UF experience, years	6.3 ± 3.0	6.7 ± 2.8	−0.71	0.48	0.16	9.8 ± 2.0	9.7 ± 1.8	0.05	0.96	0.02	8.7 ± 3.1	10.7 ± 1.1	−3.32	0.002	0.76
National team experience, years	2.0 ± 1.6	2.9 ± 2.6	−1.74	0.08	0.43	3.7 ± 3.1	5.4 ± 4.3	−1.37	0.18	0.47	4.0 ± 3.9	7.1 ± 4.0	−2.47	0.02	0.78
Weekly training sessions, ^#^	2.2 ± 0.7	2.6 ± 0.8	−1.81	0.07	0.43	1.7 ± 0.6	1.7 ± 0.5	−0.35	0.73	0.12	1.7 ± 0.6	1.4 ± 0.6	1.51	0.14	0.47
Weekly training volume, min	221 ± 76	251 ± 82	−1.63	0.11	0.38	157 ± 71	165 ± 60	−0.33	0.74	0.11	165 ± 64	122 ± 72	2.04	0.04	0.64
Country world ranking, ^#^	16.9 ± 9.6	13.0 ± 8.4	1.71	0.09	0.42	16.6 ± 9.3	9.5 ± 5.7	2.66	0.01	0.82	18.5 ± 9.2	8.4 ± 5.6	4.44	<0.001	1.19
EBUC games played, ^#^	8.8 ± 0.8	9.3 ± 1.0	−2.19	0.03	0.53	8.8 ± 0.8	8.6 ± 1.2	0.58	0.56	0.20	7.7 ± 0.7	8.2 ± 0.4	−3.21	0.003	0.82
EBUC classification, ^#^	11.1 ± 4.5	5.7 ± 3.4	5.73	<0.001	1.28	8.4 ± 3.7	4.0 ± 1.0	5.84	<0.001	1.34	7.2 ± 2.4	2.4 ± 2.0	6.55	<0.001	2.06
SOTG classification, ^#^	3.6 ± 2.1	11.0 ± 5.5	−7.04	<0.001	1.85	3.0 ± 1.9	9.6 ± 4.1	−5.25	<0.001	2.44	2.9 ± 1.7	7.1 ± 0.4	−13.21	<0.001	2.85
Rules knowledge, ^†^	2.00 ± 0.08	2.03 ± 0.08	−1.52	0.13	0.35	2.04 ± 0.13	1.97 ± 0.12	1.56	0.13	0.54	1.96 ± 0.10	1.89 ± 0.11	1.89	0.06	0.59
Fouls and body contact, ^†^	2.11 ± 0.15	1.86 ± 0.08	9.23	<0.001	1.97	2.16 ± 0.19	1.73 ± 0.25	5.79	<0.001	1.99	2.09 ± 0.11	1.98 ± 0.16	2.76	0.008	0.86
Fair-mindedness, ^†^	2.36 ± 0.15	2.07 ± 0.11	9.77	<0.001	2.17	2.49 ± 0.19	2.14 ± 0.16	5.39	<0.001	1.86	2.41 ± 0.18	2.35 ± 0.25	0.97	0.34	0.30
Positive attitude, ^†^	2.74 ± 0.33	2.35 ± 0.21	5.88	<0.001	1.37	2.81 ± 0.13	2.46 ± 0.13	7.43	<0.001	2.57	2.69 ± 0.15	2.40 ± 0.10	6.56	<0.001	2.06
Communication, ^†^	2.36 ± 0.19	2.13 ± 0.13	5.76	<0.001	1.34	2.26 ± 0.17	2.22 ± 0.20	0.55	0.58	0.19	2.16 ± 0.06	2.07 ± 0.06	4.77	<0.001	1.50
Total SOTG Score, ^‡^	11.58 ± 0.42	10.45 ± 0.32	12.49	<0.001	2.90	11.76 ± 0.30	10.53 ± 0.29	11.64	<0.001	4.02	11.31 ± 0.37	10.70 ± 0.39	5.06	<0.001	1.59
Task orientation, ^§^	4.52 ± 0.47	4.46 ± 0.42	0.55	0.58	0.13	4.25 ± 0.48	4.42 ± 0.83	−0.81	0.42	0.28	4.04 ± 0.91	4.11 ± 0.68	−0.24	0.81	0.08
Ego orientation, ^§^	3.21 ± 0.89	3.01 ± 0.89	0.95	0.35	0.22	3.11 ± 0.70	3.52 ± 0.52	−1.72	0.09	0.60	2.66 ± 0.69	3.23 ± 1.05	−2.16	0.03	0.68

SOTG (Spirit of the Game); UF (Ultimate Frisbee); EBUC (European Beach Ultimate Championship); ^#^ number, ranking or classification; ^†^ scale 0–4; ^‡^ sum of the results of the five categories; ^§^ scale 1–5.

**Table 5:** Relationship of high SOTG level with age, UF experience, national team experience, weekly training sessions and volume, country world ranking, EBUC games played and classification, and motivational climate measures, in the total sample and by competitive level.

	High SOTG Level ^†^			
		Competitive Level		
	All Players (n = 160) *r* (95%CI)	Highest (n = 74) *r* (95%CI)	Intermediate (n = 40) *r* (95%CI)	Lowest (n = 46) *r* (95%CI)
Chronological age	0.15 (−0.01; 0.30)	0.06 (−0.18; 0.29)	0.04 (−0.28; 0.36)	0.16 (−0.14; 0.44)
UF experience	−0.06 (−0.21; 0.11)	−0.09 (−0.32; 0.15)	0.00 (−0.32; 0.32)	−0.36 * (−0.60; −0.07)
National team experience	−0.18 * (−0.33; −0.02)	−0.19 (−0.40; 0.05)	−0.18 (−0.47; 0.15)	−0.35 * (−0.59; −0.05)
Weekly training sessions	−0.12 (−0.27; 0.04)	0.23 * (−0.45; 0.001) ^‡^	−0.08 (−0.39; 0.25)	0.25 (−0.06; 0.51)
Weekly training volume	−0.10 (−0.26; 0.06)	0.23 * (−0.44; 0.01) ^‡^	−0.09 (−0.40; 0.24)	0.33 * (0.03; 0.57)
Country world ranking	0.31 ** (0.14; 0.46)	0.20 (−0.06; 0.43)	0.40 * (0.05; 0.67)	0.54 ** (0.27; 0.73)
EBUC games played	−0.19 * (−0.34; −0.04)	−0.30 ** (−0.50; −0.07)	0.05 (−0.28; 0.36)	−0.39 ** (−0.62; −0.11)
EBUC classification	0.53 ** (0.40; 0.64)	0.58 ** (0.39; 0.71)	0.48 ** (0.19; 0.70)	0.65 ** (0.43; 0.79)
Task orientation	−0.05 (−0.21; 0.11)	0.09 (−0.15; 0.32)	−0.29 (−0.56; 0.04)	0.03 (−0.28; 0.32)
Ego orientation	−0.11 (−0.26; 0.05)	0.10 (−0.14; 0.33)	−0.27 (−0.54; 0.05)	−0.32 * (−0.57; −0.03)

Note: (dummy coded; regular SOTG level as the reference group). SOTG (Spirit of the Game); UF (Ultimate Frisbee); EBUC (European Beach Ultimate Championship). *r* (correlation coefficients); 95%CI (95% confidence intervals). ^†^ Dummy coded (regular SOTG level as the reference group in the analysis). ^‡^ Unacceptable collinearity (*r* > 0.98). * *p* < 0.05, ** *p* < 0.01.

**Table 6:** Summary of stepwise discriminant analyses of UF players by SOTG level (High and Regular) in the total sample and by competitive level.

	Step	Entered	WilLambda	df1	df2	df3	Exact F	df1	df2	Sig.
All players	1	EBUC classification	0.748	1	1	158	53.354	1	158	<0.001
(n = 160)	2	EBUC games played	0.679	2	1	158	37.042	2	157	<0.001
	3	Weekly training sessions	0.653	3	1	158	27.576	3	156	<0.001
Competitive Level										
Highest	1	EBUC classification	0.705	1	1	72	30.115	1	72	<0.001
(n = 74)	2	EBUC games played	0.606	2	1	72	23.059	2	71	<0.001
Intermediate	1	EBUC classification	0.720	1	1	38	14.795	1	38	<0.001
(n = 40)										
Lowest	1	EBUC classification	0.506	1	1	44	42.957	1	44	<0.001
(n = 46)										

SOTG (Spirit of the Game); UF (Ultimate Frisbee); EBUC (European Beach Ultimate Championship). Variables in the analysis: National team experience; Weekly training sessions; Weekly training volume; Country world ranking; EBUC games played; EBUC classification; Task orientation; Ego orientation.
